# Time-to-Event Analysis of Factors Influencing Delay in Discharge from a Subacute Complex Discharge Unit during the First Year of the Pandemic (2020) in an Irish Tertiary Centre Hospital

**DOI:** 10.3390/healthcare11040627

**Published:** 2023-02-20

**Authors:** Nithya Rajendran, Puteri Maisarah Rameli, Keneilwe Malomo, Declan Byrne, Joseph Browne, Ontefetse Ntlholang

**Affiliations:** 1 Department of General Internal Medicine, St. James’s Hospital, James’s Street, D08 NHY1 Dublin, Ireland; 2 Department of Geriatrics and General Internal Medicine, St. James’s Hospital, James’s Street, D08 NHY1 Dublin, Ireland

**Keywords:** complex discharge unit, Cox regression analysis, COVID-19, delayed transfers of care, Kaplan–Meier analysis, length of stay, pandemic, time-to-event analysis, discharge delay

## Abstract

Our study aimed to analyse delaying factors amongst patients with a length of stay (LOS) > 15 days during the COVID-19 pandemic using time-to-event analysis. A total of 390 patients were admitted between March 2020–February 2021 to the subacute complex discharge unit in St James’s Hospital: 326 (83.6%) were >65 years of age and 233 (59.7%) were female. The median (IQR) age was 79 (70–86) years with a median (IQR) of 19.4 (10–41) days. A total of 237 (60.7%) events were uncensored, with LOS > 15 days, of which 138 (58.2%) were female and 124 (52.32%) had >4 comorbidities; 153 (39.2%) were censored into LOS ≤ 15 days, and death occurred in 19 (4.8%). Kaplan–Meier’s plot compared factors causing a delay in discharge to the single factors: age, gender, and multimorbidity. A multivariate Cox regression analysis adjusted to age, gender, and multimorbidity predicted factors affecting LOS. Further research is required to explore multimorbidity as a risk factor for mortality in patients with prolonged LOS within a complex discharge unit and target gender-specific frailty measures to achieve high-quality patient management.

## 1. Introduction

Length of stay is the most common outcome measure of effectiveness in discharge planning [1]. It refers to the time expressed in days between admission to and discharge from the hospital [2]. Worldwide, variability in the measure of delayed discharges based on hospital stay exists. Countries such as the Netherlands define delayed discharges as a length of stay exceeding 50% of the average length of stay for the general population, in the previous calendar year. In countries such as Australia and Singapore, a delayed discharge is defined as length of stay exceeding 21 days and 35 days, respectively. A notional application of the Dutch model to the hospital inpatient enquiry dataset provides estimates of possible delayed discharge, but does not define the delayed length of stay days in Ireland [3]. During the COVID-19 pandemic, the average inpatient length of stay was 5.8 days, representing a slight increase in the average inpatient length of stay of 5.7 days between 2016–2019 [2].

Tackling delays in patient discharge is an ongoing challenge, and with the emergence of the COVID-19 pandemic, there has been a greater strain on the Irish healthcare system. Subacute complex discharge units (CDU) are a subset of intermediate care that provides person-centred planning [4] through an individualised, integrated care pathway to improve functional outcomes and reduce the length of stay [5]. Many countries do not have a specially dedicated CDU, with the term most often interchangeably used with a transitional care unit instead. Commonly located on the hospital premises, CDU assist in the transition of medically complex patients post-acute care to their homes or long-term residential places. A dedicated multidisciplinary team comprising allied health professionals and a medical team specialised in gerontology and complex discharge provides care in a nurturing environment through subacute rehabilitation of medically complex patients who have become deconditioned throughout their inpatient stay. Furthermore, the CDU also assists in the organisation of community social support before patient discharge from the hospital [6].

Other factors to consider are defined under delayed transfers of care: delayed discharges awaiting discharge home, community care provision through the Home Support Service, or placement or transfer to long-term residential places. Formerly, a delayed discharge was described as a patient deemed medically fit for discharge from an acute bed who remained in the hospital because they were awaiting support or care following discharge [7]. The emergence of the COVID-19 pandemic has caused significant disruptions in discharge planning, discontinuity, and incoordination between subacute CDU and community care [4] due to service curtailment and limited step-down care options [8].

Previous literature findings linked to discharge delays discuss multimorbidity [9], cognitive deterioration [5], and frailty [10] as the leading causes of discharge delays. Complex multimorbidity is described as the co-occurrence of three or more chronic medical conditions involving at least three systems in the body [9]. Frailty [10] and multimorbidity [11] are the most common causes of delayed discharge among older adults leading to poor functional outcomes. There is also a significant correlation between cognitive impairment and dependency on the overall length of stay [12]. Moreover, patient-centred needs comprising cognitive dysfunction, psychosocial needs, carer involvement, and community services linked to Home Support Services have also been described [5]. Prolonged hospital stays are known to have a detrimental impact on patient health through increased risk of deconditioning, healthcare-associated infections, and mortality [2].

Delayed discharges have serious implications on healthcare costs. According to the Health Service Executive Performance report produced in 2020, delayed transfer of care was notable in 11.6% of patients with complex needs. Furthermore, 5.2% of patients had rehabilitation needs leading to delays in the transfer of care. Several elements of clinical practice were affected during the first year of the COVID-19 pandemic, including interrupted regular management of medical conditions contributing to complications of comorbid disease and physical follow-up appointments replaced with virtual assessments, limiting clinical examination and access to rehabilitation. Likewise, the delays in surgical procedures and chemotherapy in individuals with malignancy lead to potential complications, consequently influencing patient rehabilitation potential. Our study aimed to analyse factors causing delays in discharge from a subacute CDU during the first year of the COVID-19 pandemic where clinical practice experienced a drastic change owing to the initial period of confinement [13].

## 2. Materials and Methods

The authors gained approval from the St James’s Hospital Research & Innovation Office (reference number: 7484 on 9 February 2022) and St James’s/Tallaght University Hospital Research & Ethics Office (submission number: 717 on 11 February 2022) prior to the study taking place.

### 2.1. Setting

This study was conducted in St James’s Hospital, the largest acute academic teaching model 4 hospital in Ireland, based in the south inner city of Dublin. Within St James’s Hospital, there is a 23-bedded CDU that coordinates the safe and effective discharge of medically stable patients. A multidisciplinary team comprising of allied health professionals (physiotherapist, occupational therapist, social worker, speech and language therapist, clinical nutritionist) and a medical team specialised in gerontology and complex discharge (consultant physician, registrar, senior house officer, and intern) identifies medically complex patients who are stable and nearing discharge and transfers them to the CDU. Through early goal-oriented input from the multidisciplinary team, it is envisaged that the length of stay for all patients within the CDU does not exceed six weeks. This retrospective, cross-sectional study provides valuable insight into the factors contributing to the delayed discharges of patients from a subacute CDU, especially during the first year of the COVID-19 pandemic.

### 2.2. Data Sources

All information was sourced directly from the hospital database via electronic patient records. We focused on all adult patients that were admitted to CDU between March 2020–February 2021 of the pandemic, with confirmed hospital length of stay > 15 days. Two independent reviewers extracted and extensively evaluated the collected data for suitability using an excel sheet. All information was anonymised before they were further stratified according to age, gender, primary diagnoses, the prevalence of multimorbidity, corresponding length of stay, and causes for delayed discharges from the CDU Multimorbidity was categorised using the Charlson comorbidity index, which helps in predicting the mortality risk dependent on the number of comorbidities that a patient has [14]. In our study, integer 0 was assigned to all patients with no comorbidity and 1 was assigned for each comorbid disease. All details pertaining to delayed discharges were extracted directly from inpatient notes and medical discharge summaries. This study was reported in accordance with Strengthening the Reporting of Observational Studies in Epidemiology (STROBE) guidelines (Supplementary File S3) [15].

### 2.3. Statistical Analyses

All statistical analyses were conducted using a statistical analysis software platform: IBM SPSS, version 29. The comparative analysis of length of stay between March 2020–February 2021 was performed using Kaplan–Meier for each factor: age, gender, multimorbidity, and common reasons for delayed transfers of care. Further analyses with Cox regressions using multiple covariates were used to identify factors causing delays in discharge during the period examined. In the main stratum, age was subdivided into four groups: <65 years of age, between 65–75 years of age, 75–85 years of age, and >85 years of age; gender was sub-classified into male and female; and multimorbidity was stratified into sample A ≤ 4 comorbid diseases and B > 4 comorbidities. Patients were compared and analysed by survival analysis; the Kaplan–Meier method was used for single-factor comparison, and the Cox regression model was used for multifactor comparison. The event of interest coded with the value of 1 in our study was delayed discharge; patients with a length of stay < 15 days and any deaths were assigned a value of 0 (right censored events). Our main objective was to predict a delay in discharge. With the standard Cox regression model, we encountered violations of proportional hazard. Therefore, we used a stratified Cox model allowing for covariates with nonproportional hazard; stratum: age, gender, and multimorbidity.

## 3. Results

A total of 390 patients were admitted between March 2020–February 2021 to the CDU of St James’s Hospital. Among these, 326 (83.6%) patients were >65 years of age with a median age (IQR) of 79 (70–86) years. The gender distribution of men and women was 40.3% vs. 59.7%, respectively; 188 (48.20%) patients had >4 comorbidities. For our study, we defined prolonged length of stay as a hospital length of stay > 15 days. During the COVID-19 pandemic, the CDU median (IQR) length of stay was 19.4 (10–41) days. The most common primary diagnosis on admission was infection or sepsis (23.33%). Each stratum was preliminarily analysed using the Mann–Whitney test and Chi-square (gender) respectively. (Table 1).

Following this, we proceeded with time-to-event analysis. Kaplan–Meier’s plot allowed for the comparison of factors causing a delay in discharge to single factors: age, gender, and multimorbidity. A total of 237 (60.7%) events were uncensored, with a length of stay > 15 days, of which 138 (58.2%) were female and 124 (52.32%) had >4 comorbidities; 153 (39.2%) were censored into a length of stay ≤ 15 days, and death occurred in 19 (4.8%). The Kaplan–Meier method allowed for a comparison of five major factors responsible for delays in discharge from the CDU: complications arising from primary diagnoses or multimorbidity; healthcare-associated infections such as COVID infection, hospital- acquired pneumonia, and catheter-associated infections; frailty and/or falls necessitating integrated rehabilitation; patient-centred needs consisting of cognitive decline from baseline, psychosocial requirements, and carer involvement; and community services, against the length of stay.

**Figure 1:**
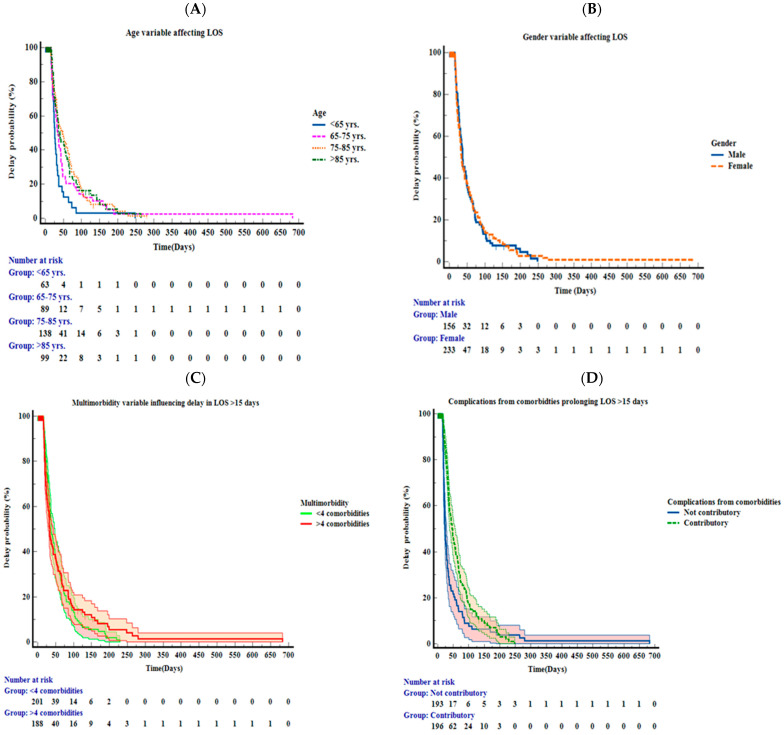

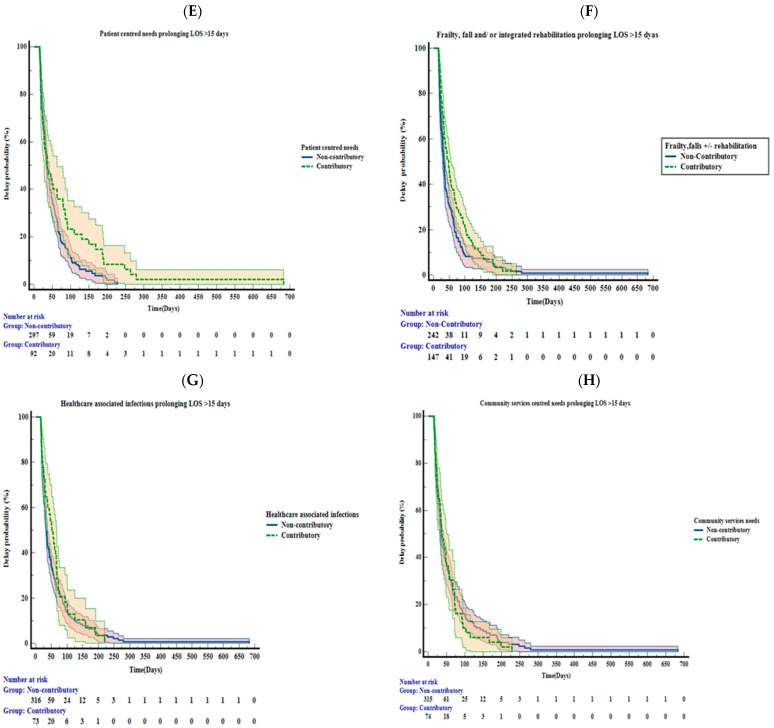
Kaplan–Meier curves for (**A**) age, (**B**) gender, (**C**) multimorbidity, (**D**) complications arising from primary diagnoses, (**E**) healthcare-associated infections, (**F**) frailty or falls necessitating integrated rehabilitation, (**G**) patient-centred needs, (**H**) community services contributing to delays in discharge during the first year of the pandemic in complex discharge unit. Figure legend: Each figure exhibits the overall delay in the length of stay (LOS) in number of days, with the numbers of patients at risk respectively below the curve.

In our study, we observed how more female than male patients experienced delays in their length of stay within the CDU (Figure 1B). The mean age of patients with a delayed length of stay was 77.51 years compared to those with a normal length of stay with a mean age of 75.25 years. Patients who were between 65–75 years of age were more likely to experience prolonged lengths of stay (Figure 1A). Furthermore, patients with >4 comorbidities were most likely to experience a delay in the length of stay exceeding >15 days (Figure 1C). Complications arising directly from comorbidities were not seen to contribute to prolonging the length of stay > 15 days. The most common complications arising from comorbidities were cardiovascular causes such as arrhythmias (15.8%), left ventricular dysfunction (20%), and hypertension (25.3%); neurological causes such as cognitive impairment secondary to infection (17.2%) and worsening of Parkinson’s disease (8.78%); and respiratory causes such as exacerbation of airway disease (4.8%). Endocrine complications accounted for 8.1% of patients with diabetes mellitus and thyroid disorders (Figure 1D). Patients whose inpatient stays were seen to be complicated by healthcare-associated infections were not observed to have prolonged lengths of stay > 15 days (Figure 1E). Additionally, frailty, falls, and/or integrated rehabilitation or community services did not result in a prolonged length of stay > 15 days. (Figure 1F,H) Patient-centred needs were a significant contributing factor to a prolonged length of stay > 15 days (Figure 1G). Our univariate KM plot analysis showed statistically significant results *p* < 0.05 for age and factors such as complications that arose from comorbidities, frailty, or falls necessitating integrated rehabilitation and patient-centred needs.

Using stratified Cox regression analysis, we analysed the strata of age, gender, and multimorbidity against the five factors associated with delay in discharge (Table 2). The five main factors discussed were not responsible for the delayed length of stay in patients > 85 years of age. In a comparison of each stratum to the five main factors causing a delay in discharge, it was noted that complications arising from primary diagnoses and patient-centred needs had statistically significant results *p* < 0.05.

This analysis predicted factors affecting the length of stay within the CDU: Age strata of 65–75 years of age and 75–85 years of age [HR 0.233; 95% CI (0.077–0.708); *p* = 0.010] and [HR 0.301; 95% CI (0.155–0.588); *p* < 0.001] had a common factor of patient-centred needs prolonging the length of stay. Interestingly, both genders and individuals with ≤4 comorbid diseases and >4 comorbidities had complications arising from primary diagnoses and patient-centred needs prolonging the length of stay with *p* < 0.05, which are statistically significant results. The common factors complications arising from comorbidities in males [HR 0.145; 95% CI (0.081–0.261); *p* < 0.001] and females [HR 0.479; 95% CI (0.311–0.737); *p* < 0.001]; and patient-centred needs in males [HR 0.472; 95 % CI (0.243–0.917); *p* 0.027] and females [HR 0.361; 95% CI (0.215–0.608); *p* < 0.001] exhibited statistically significant results.

Interestingly, we noted that the presence of multimorbidity in patients < 65 years of age exhibited statistically significant results influencing length of stay > 15 days [HR 0.097; 95% CI (0.028–0.340); *p* < 0.001] in contrast to patients > 85 years of age [HR 0.539; 95% CI (0.272–1.065); *p* = 0.075]. The reasoning for this could likely be due to increased mortality in those > 85 years of age (patient deaths within the CDU were excluded from this study) and a steady drop in attendance to the hospital due to fear of acquiring COVID-19 infection. Several patients < 65 years of age were found to have new medical diagnoses that complicated their inpatient course, warranting involvement from other specialties regarding management, thereby affecting the length of stay. Factors such as frailty or patient-centred needs were observed to affect the length of stay for women (*p* < 0.001), but not in men (*p* = 0.286). This is consistent with previous literature reports of a higher prevalence of frailty in women (Supplementary File Figures S1 and S2).

## 4. Discussion

With the emergence of the COVID-19 pandemic, several challenges were witnessed. Substantial changes were made in clinical management, particularly within the first year of the COVID-19 pandemic. Several routine elective surgeries and physical outpatient follow-up appointments were cancelled and public health advice on isolation precautions resulted in a decline in admission rates. There was also an increased caseload on the country’s health system [16,17]. Our retrospective cross-sectional analysis of patients admitted within the first year of the COVID-19 pandemic to a subacute CDU delineated five factors responsible for delays in discharge.

First, it was evident that the COVID-19 pandemic resulted in many patients exceeding the average length of stay in the hospital, with a subsequent impact on transfers of care. Within our CDU, 37.94% of patients experienced a length of stay exceeding > 15 days as a result of complications arising from primary diagnoses or multimorbidity. This was followed by 25.38% of patients due to frailty or falls necessitating integrated rehabilitation, 14.35% of patients consequent to issues surrounding community services provision, 13.07% due to patient-centred needs, and finally 10.52% of patients experiencing a length of stay exceeding > 15 days due to healthcare-associated infections. Prior to the COVID-19 pandemic, we found only one study from our CDU centre describing the association between clinical frailty scoring measures and length of stay. Furthermore, the study concluded the assessment of only one factor contributing to complex discharge. The majority of admissions to the CDU prior to the COVID-19 pandemic were due to falls, with moderately frail patients observed to have longer lengths of stay [18]. These findings were different to our observation of the CDU during the COVID-19 pandemic whereby complications arising from primary diagnoses or multimorbidity were acknowledged as major factors for delayed length of stay.

When comparing our patient demographics to the current literature, we noted that the distribution of age and gender did not vary significantly before and during the COVID-19 pandemic. Many patients admitted to our CDU were female, predominantly > 65 years of age, and affected by three factors common to delaying the length of stay within their age group. These factors were complications arising from primary diagnoses or multimorbidity, frailty necessitating integrated rehabilitation, and patient-centred needs [19]. Our findings were similar to a recently published Singaporean study that showed how continuity of integrated care in the community was considerably affected during the COVID-19 pandemic. In their study, the authors discuss the reluctance or hesitancy from both formal and informal caregivers to accept community services due to fear of contracting COVID-19 infection. This resulted in many patients experiencing subsequent delayed lengths of stay [20]. Another study from the United States made similar observations when evaluating the discharge processes in a skilled nursing facility [21]. The pressured situation observed in social care, especially long-term care facilities, is something that all hospitals are internationally familiar with and continuously struggle to find resolutions.

In our study, patient-centred needs remained the most prevalent factor affecting the length of stay against the strata of age, gender, and multimorbidity. Notably, abrupt changes during the initial period of the pandemic increased social isolation in older adults, resulting in a negative impact on cognitive function [22]. The psychosocial impact on the carer and their involvement in providing care became apparent during the pandemic. With the increase in COVID-19 prevalence, if a carer acquired COVID infection, difficulties arose when planning for discharge, which is of relevance to older adults as isolation facilities were not designated for older populations dependent on the carer. Another aspect involved in the patient-centred approach is patient preference on discharge planning, and this was not an option due to the challenges posed by COVID-19 [23]. Further research and studies are required to deduce appropriate measures to improve patient-centred requirements, particularly in the event of a future pandemic.

We acknowledge that the occurrence of multimorbidity is associated with much higher rates of admission to the hospital and subsequent readmission [24]. Through the utilisation of the Charlson comorbidity index scoring, we subcategorised complex multimorbidity as follows: ≤4 as mildly to moderately complex, and those with 5 or more as highly complex multimorbidity [25]. In our analysis, we were able to ascertain the association of multimorbidity with length of stay. Complications arising directly from multimorbidity were seen as a noncontributory factor in prolonging the length of stay > 15 days. Interestingly, patients that were admitted to the CDU with ≤4 comorbid diseases and >4 comorbidities experienced the same delaying factors for length of stay, including complications arising from primary diagnoses or multimorbidity and patient-centred needs. This key finding reiterates how physicians should adopt a targeted approach, addressing comorbidity individually instead of treating it as a single comorbid disease [26]. We would recommend using this strategy as a tool in discharge planning healthcare models for effective patient management.

There is a higher prevalence of frailty in women due to oestrogen dysfunction and associated changes influencing their health [27]. Another aspect worth promulgating in discharge planning would be the inclusion of gender-specific frailty measures that would have an impact on fall prevention and rehabilitation, therefore reducing the length of stay. During the initial period of the pandemic, community services witnessed reduced staffing, a decline in logistics, care facilities with reduced services, and very few referrals to long-term care were accepted if deemed necessary [28]. More research must be applied to the needs of patients with complex care needs and their informal helpers or networks that emerged during the pandemic.

Although healthcare-associated infections are a major determinant in delaying discharge, the incidence of infections such as hospital-acquired pneumonia and catheter-related infections has seen a significant decline with strict hygiene precautions. The opposite was true for COVID-19 infection during the first year of the pandemic, whereby many hospitals, including our CDU, observed high rates of transmission of COVID-19 infections despite adequate contact precautions and staff training. Protracted hospitalisation became commonplace, as many patients found themselves unfortunately having to remain in the hospital to complete 14 days of isolation before discharge home or to their long-term residential place [29]. Delayed length of stay as a result of COVID-19 infection is now improving with the evolving nature of the pandemic, fewer days of isolation advised by national guidelines, and the availability of more isolation facilities for clinically stable COVID-19 patients.

## 5. Strengths and Limitations

To our knowledge, this is the first study that has investigated the time-to-event analysis of factors influencing delayed discharges exclusively in complex patients admitted to a CDU during the COVID-19 pandemic. The electronic patient record system used for the study allowed for ease in traceability and transparency of all data. Nevertheless, there were some limitations to the study. Our study was conducted in a single centre explicitly focusing on delaying factors as opposed to factors leading to readmission. The study solely focused on patients admitted to the CDU instead of incorporating all hospital admissions and readmissions during the COVID-19 pandemic. Third, the interpretation of our findings is limited to the sample size; therefore, the extrapolation and application of these data may differ across other CDUs or relevant centres. Finally, mortality and readmission were not explored in detail during this study.

## 6. Conclusions

Our study is the first to analyse common factors delaying discharge from the CDU such as complexities arising from primary diagnoses and patient-centred needs such as cognitive decline, psychosocial needs, and carer needs. The analysis of these factors associated with delays in discharge during the pandemic can aid in medical decisions and form the framework for future contingency planning on resources should we encounter another pandemic. We were unable to draw inferences from a previous study in the pre-pandemic period, where frailty and frailty scale measures influencing length of stay were studied extensively. Further research is required to explore multimorbidity as a risk factor for mortality in patients with prolonged lengths of stay within a CDU and targeted gender-specific frailty measures to achieve high-quality patient management. Additionally, more studies on analysing associated patient-centred needs would provide excellent information to develop effective strategies for complex discharge planning.

## Figures and Tables

**Table 1:** Strata qualitatively assessed using Mann–Whitney and Chi-square (gender) respectively for patients admitted to the complex discharge unit.

	2020 (*n* = 390)		
Patient Characteristics	LOS ≤ 15 days (*n* = 153)	LOS > 15 days (*n* = 237)	*p* <0.05 Statistical significance
Age (Mean ± SD)	75.25 ± 12.50	77.51 ± 12.62	*p* = 0.038
Gender: Female Male	62.09% 37.90%	58.22% 41.77%	*p* = 0.51 (Yates correction to avoid type 1 error); *p* = 0.46
Multimorbidity: ≤4 >4	15 64	99 124	*p* < 0.05

LOS, length of stay.

**Table 2:** Variables independently associated with delay in discharge (LOS, length of stay >15 days) from stratified Cox regression analysis.

Chief Characteristics		Covariates	B Coefficient	Hazard Ratio (95% CI)	*p* Value
Age	<65	Complications/comorbidities prolonging LOS	−2.329	0.097 (0.028–0.340)	<0.001
		Healthcare-associated infection	−1.005	0.366 (0.095–1.416)	0.146
		Frailty, falls, and/or integrated rehabilitation needs	−0.063	0.939 (0.272–3.239)	0.921
		Patient-centred needs	−0.039	0.962 (0.325–2.850)	0.944
		Community services	−0.216	0.806 (0.321–2.020)	0.645
Age	65–75	Complications/comorbidities prolonging LOS	−0.759	0.468 (0.206–1.065)	0.070
		Healthcare-associated infection	0.190	1.209 (0.506–2.887)	0.669
		Frailty, falls, and/or integrated rehabilitation needs	−0.893	0.410 (0.211–0.795)	0.008
		Patient-centred needs	−1.455	0.233 (0.077–0.708)	0.010
		Community services	0.67	1.069 (0.522–2.188)	0.855
Age	75–85	Complications/comorbidities prolonging LOS	−1.270	0.281 (0.160–0.492)	<0.001
		Healthcare-associated infection	−0.628	0.533 (0.288–0.987)	0.045
		Frailty, falls, and/or integrated rehabilitation needs	−0.473	0.623 (0.387–1.002)	0.051
		Patient-centred needs	−1.199	0.301 (0.155–0.588)	<0.001
		Community services	−0.075	0.928 (0.536–1.605)	0.789
Age	>85	Complications/comorbidities prolonging LOS	−0.619	0.539 (0.272–1.065)	0.075
		Healthcare-associated infection	−0.177	0.838 (0.369–1.904)	0.673
		Frailty, falls, and/or integrated rehabilitation needs	−0.213	0.808 (0.427–1.531)	0.514
		Patient-centred needs	−0.498	0.608 (0.262–1.408)	0.246
		Community services	−0.203	0.817 (0.394–1.693)	0.586
Gender	Male	Complications/comorbidities prolonging LOS	−1.930	0.145 (0.081–0.261)	<0.001
		Healthcare-associated infection	−0.574	0.563 (0.314–1.010)	0.054
		Frailty, falls, and/or integrated rehabilitation needs	−0.235	0.790 (0.513–1.217)	0.286
		Patient-centred needs	−0.751	0.472 (0.243–0.917)	0.027
		Community services	−0.027	0.973 (0.621–1.524)	0.905
Gender	Female	Complications/comorbidities prolonging LOS	−0.736	0.479 (0.311–0.737)	<0.001
		Healthcare-associated infection	−0.157	0.854 (0.524–1.393)	0.528
		Frailty, falls, and/or integrated rehabilitation needs	−0.521	0.594 (0.401–0.880)	0.009
		Patient-centred needs	−1.018	0.361 (0.215–0.608)	<0.001
		Community services	−0.073	0.930 (0.520–1.663)	0.809
Multi morbidity	≤4	Complications/comorbidities prolonging LOS	−0.858	0.424 (0.267–0.672)	<0.001
		Healthcare-associated infection	−0.351	0.704 (0.411–1.207)	0.202
		Frailty, falls, and/or integrated rehabilitation needs	−0.843	0.431 (0.285–0.651)	<0.001
		Patient-centred needs	−0.828	0.437 (0.247–0.772)	0.004
		Community services	−0.234	0.792 (0.501–1.252)	0.317
Multi morbidity	>4	Complications/comorbidities prolonging LOS	−1.179	0.308 (0.190–0.497)	<0.001
		Healthcare-associated infection	−0.086	0.917 (0.551–1.528)	0.740
		Frailty, falls, and/or integrated rehabilitation needs	−0.256	0.774 (0.512–1.170)	0.224
		Patient-centred needs	−1.045	0.352 (0.192–0.644)	<0.001
		Community services	0.196	1.211 (0.739–2.002)	0.442

## Data Availability

All data relevant to the study are included in the article or uploaded as supplementary information.

## Supplementary Materials

The following supporting information can be downloaded at https://www.mdpi.com/article/10.3390/healthcare11040627/s1, Figure S1: Forest plot depicting age and gender strata-associated hazard ratio, Figure S2: Forest plot depicting multimorbidity (MM) strata-associated hazard ratio. Supplementary File S3: STROBE checklist.
