# Supplementary material for: Time-to-Event Analysis of Factors Influencing Delay in Discharge from a Subacute Complex Discharge Unit during the First Year of the Pandemic (2020) in an Irish Tertiary Centre Hospital
Source: Healthcare (Basel). 2023 Feb 20;11(4):627. doi: 10.3390/healthcare11040627 (PMC9956250; doi:10.3390/healthcare11040627)
Supplement: Supplementary file 1 [file healthcare-11-00627-s001.zip › Supplementary File S3-STROBE-checklist.pdf]

STROBE Statement—checklist of items that should be included in reports of observational studies

	Item No	Recommendation
**Title and abstract**	1	(*a*) Indicate the study's design with a commonly used term in the title or the abstract<br>Retrospective observational study as stated in the Abstract, Materials and Methods sections
		(*b*) Provide in the abstract an informative and balanced summary of what was done and what was found<br>Provided in the Abstract page1
**Introduction**		
Background/rationale	2	Explain the scientific background and rationale for the investigation being reported<br>Included in Introduction section page 1-2
Objectives	3	State specific objectives, including any prespecified hypotheses<br>Included in the Introduction section page 1-2
**Methods**		
Study design	4	Present key elements of study design early in the paper<br>Included in the Materials and Methods section page 2-3
Setting	5	Describe the setting, locations, and relevant dates, including periods of recruitment, exposure, follow-up, and data collection<br>Included in the Materials and Methods section page 2-3
Participants	6	(*a*) *Cohort study*—Give the eligibility criteria, and the sources and methods of selection of participants. Describe methods of follow-up<br>*Case-control study*—Give the eligibility criteria, and the sources and methods of case ascertainment and control selection. Give the rationale for the choice of cases and controls<br>*Cross-sectional study*—Give the eligibility criteria, and the sources and methods of selection of participants<br>Included in the Materials and Methods section page 2- 3
		(*b*) *Cohort study*—For matched studies, give matching criteria and number of exposed and unexposed<br>*Case-control study*—For matched studies, give matching criteria and the number of controls per case<br>Not applicable
Variables	7	Clearly define all outcomes, exposures, predictors, potential confounders, and effect modifiers. Give diagnostic criteria, if applicable<br>Included in the Materials and Methods section page 2-3
Data sources/ measurement	8*	For each variable of interest, give sources of data and details of methods of assessment (measurement). Describe comparability of assessment methods if there is more than one group<br>Included in the Materials and Methods section page 2-3
Bias	9	Describe any efforts to address potential sources of bias<br>Addressed in the Strengths and Limitations section page 10
Study size	10	Explain how the study size was arrived at<br>Included in the Materials and Methods section page 2-3
Quantitative variables	11	Explain how quantitative variables were handled in the analyses. If applicable, describe which groupings were chosen and why

		Included in the Materials and Methods section page 2-3
Statistical methods	12	(*a*) Describe all statistical methods, including those used to control for confounding Included in the Materials and Methods section page 2-3
		(*b*) Describe any methods used to examine subgroups and interactions Included in the Materials and Methods section page 2-3
		(*c*) Explain how missing data were addressed Not applicable
		(*d*) *Cohort study*—If applicable, explain how loss to follow-up was addressed<br>*Case-control study*—If applicable, explain how matching of cases and controls was addressed<br>*Cross-sectional study*—If applicable, describe analytical methods taking account of sampling strategy Included in the Materials and Methods section page 2-3
		(*e*) Describe any sensitivity analyses Not applicable

Continued on next page

**Results**		
Participants	13*	(a) Report numbers of individuals at each stage of study—eg numbers potentially eligible, examined for eligibility, confirmed eligible, included in the study, completing follow-up, and analysed Included in the Results section page 3-9
		(b) Give reasons for non-participation at each stage Not applicable
		(c) Consider use of a flow diagram Not required
Descriptive data	14*	(a) Give characteristics of study participants (eg demographic, clinical, social) and information on exposures and potential confounders Included in the Results section page 3-9
		(b) Indicate number of participants with missing data for each variable of interest Not applicable
		(c) *Cohort study*—Summarise follow-up time (eg, average and total amount) Included in the Results section page 3-9
Outcome data	15*	*Cohort study*—Report numbers of outcome events or summary measures over time
		Case-control study—Report numbers in each exposure category, or summary measures of exposure
		Cross-sectional study—Report numbers of outcome events or summary measures Included in the Results section page 3-9
Main results	16	(*a*) Give unadjusted estimates and, if applicable, confounder-adjusted estimates and their precision (eg, 95% confidence interval). Make clear which confounders were adjusted for and why they were included Included in the Results section page 3-9
		(*b*) Report category boundaries when continuous variables were categorized Included in the Results section page 3-9
		(*c*) If relevant, consider translating estimates of relative risk into absolute risk for a meaningful time period
Other analyses	17	Report other analyses done—eg analyses of subgroups and interactions, and sensitivity analyses Not applicable
**Discussion**		
Key results	18	Summarise key results with reference to study objectives Included in the Discussion page 9-10
Limitations	19	Discuss limitations of the study, taking into account sources of potential bias or imprecision. Discuss both direction and magnitude of any potential bias Included in the Strengths and Limitations section page 9-10
Interpretation	20	Give a cautious overall interpretation of results considering objectives, limitations, multiplicity of analyses, results from similar studies, and other relevant evidence Included in the Discussion page 9-10
Generalisability	21	Discuss the generalisability (external validity) of the study results Included in the Discussion page 9-10
**Other information**		
Funding	22	Give the source of funding and the role of the funders for the present study and, if applicable, for the original study on which the present article is based Provided in text page 11

*Give information separately for cases and controls in case-control studies and, if applicable, for exposed and unexposed groups in cohort and cross-sectional studies.

Note: An Explanation and Elaboration article discusses each checklist item and gives methodological background and published examples of transparent reporting. The STROBE checklist is best used in conjunction with this article (freely available on the Web sites of PLoS Medicine at http://www.plosmedicine.org/, Annals of Internal Medicine at http://www.annals.org/, and Epidemiology at http://www.epidem.com/). Information on the STROBE Initiative is available at www.strobe-statement.org.

Signed P.M. Rameli

08/02/2023
