# Supplementary figures and images for: Time-to-Event Analysis of Factors Influencing Delay in Discharge from a Subacute Complex Discharge Unit during the First Year of the Pandemic (2020) in an Irish Tertiary Centre Hospital
Source: Healthcare (Basel). 2023 Feb 20;11(4):627. doi: 10.3390/healthcare11040627 (PMC9956250; doi:10.3390/healthcare11040627)

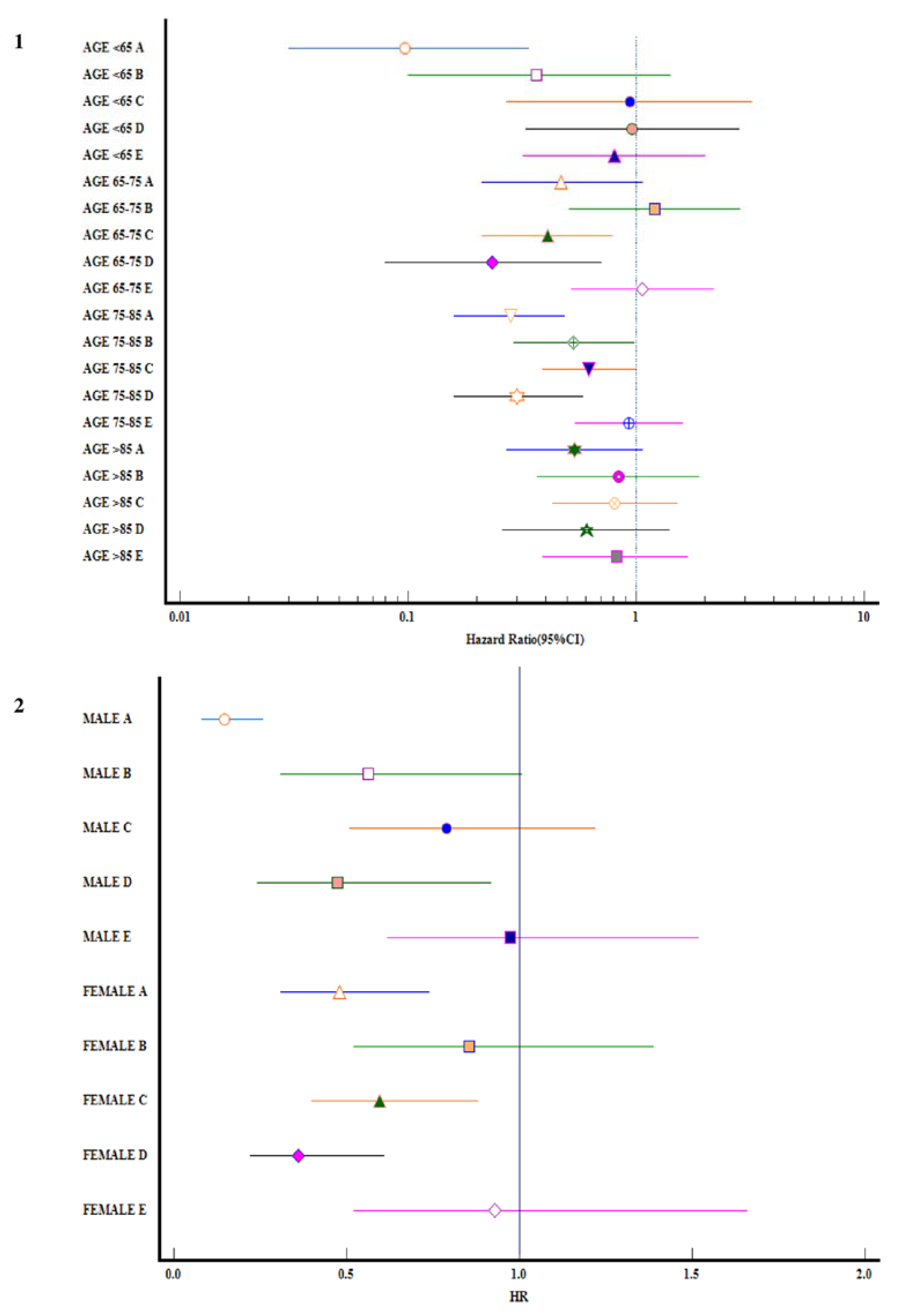

Supplement: Supplementary file 1 [file healthcare-11-00627-s001.zip › Figure S1.tif]

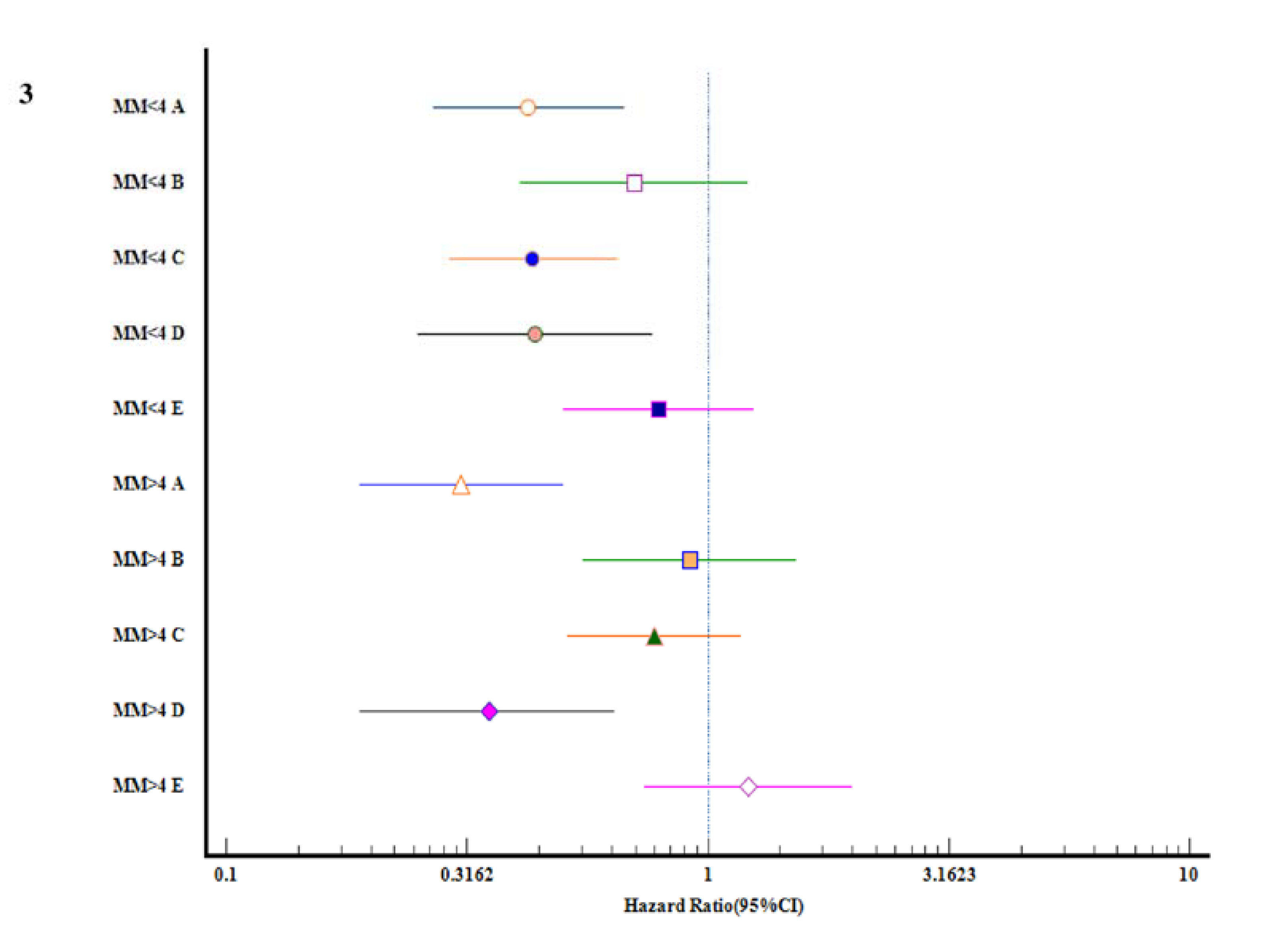

Supplement: Supplementary file 1 [file healthcare-11-00627-s001.zip › Figure S2.tif]
